# Ovulation induction and subfertile untreated conception groups offer improved options for interpreting risks associated with ART

**DOI:** 10.1007/s10815-024-03060-6

**Published:** 2024-03-12

**Authors:** Michele Hansen, Roger J. Hart, Elizabeth Milne, Carol Bower, Melanie L. Walls, John L. Yovich, Peter Burton, Yanhe Liu, Hamish Barblett, Anna Kemp-Casey

**Affiliations:** 1 Telethon Kids Institute, UWA Centre for Child Health Research, University of Western Australia, Perth, Australia; 2 Division of Obstetrics and Gynecology, University of Western Australia, Perth, Australia; 3 Fertility Specialists of Western Australia, and City Fertility Australia, Perth, Australia; 4 PIVET Medical Centre, Perth, Australia; 5 School of Pharmacy and Biomedical Sciences, Curtin University, Perth, Australia; 6 Concept Fertility Centre, Perth, Australia; 7 Fertility North, Perth, Australia; 8 School of Human Sciences, University of Western Australia, Perth, Australia; 9 Genea Hollywood Fertility, Perth, Australia; 10 Quality Use of Medicines and Pharmacy Research Centre, University of South Australia, Adelaide, Australia

**Keywords:** Assisted reproductive technology, IVF, Subfertility, Ovulation induction, ART outcomes

## Abstract

**Purpose:**

To identify and characterise appropriate comparison groups for population studies of health outcomes in ART-conceived births: ovulation induction (OI), subfertile untreated and fertile natural conceptions. Our secondary objective was to examine whether known risks of pregnancy complications and adverse birth outcomes in ART births are elevated in comparison with subfertile (untreated and OI) conception groups.

**Methods:**

We linked State and Commonwealth datasets to identify all live and stillbirths (≥ 20 weeks) in Western Australia from 2003 to 2014 by method of conception. Demographic characteristics, maternal pre-existing conditions, adverse obstetric history and pregnancy complications were compared across conception groups. Generalised estimating equations were used to estimate adjusted risk ratios (aRRs) and 95% confidence intervals (CI) for pregnancy complications and birth outcomes in singletons.

**Results:**

We identified 9456 ART, 3870 OI, 11,484 subfertile untreated and 303,921 fertile naturally conceived deliveries. OI and subfertile untreated groups more closely resembled the ART group than the fertile group; however, some differences remained across parity, maternal age, pre-existing conditions and obstetric history. In multivariate analyses, ART singletons had greater risks of placental problems (e.g. placenta praevia aRR 2.42 (95% CI 1.82–3.20)) and adverse birth outcomes (e.g. preterm birth aRR 1.38 (95% CI 1.25–1.52)) than the subfertile untreated group, while OI singletons were more similar to the subfertile group with higher risk of preeclampsia and gestational diabetes.

**Conclusion:**

OI and subfertile untreated conception groups offer improved options for interpreting health outcomes in ART births. Pregnancy complications (particularly placental disorders) and adverse outcomes at delivery are more common following ART.

**Supplementary Information:**

The online version contains supplementary material available at 10.1007/s10815-024-03060-6.

## Introduction

It is well established that pregnancies achieved through assisted reproductive technology (ART), defined as the in vitro manipulation of eggs and sperm using standard IVF or intracytoplasmic sperm injection (ICSI) and the transfer of fresh or frozen-thawed embryos, have a greater risk of pregnancy complications and poor perinatal outcomes than non-ART pregnancies [1, 2]. Pregnancy complications such as gestational hypertension and diabetes [1, 3], placental problems [1, 4] and adverse perinatal outcomes including preterm birth [1], low birth weight [1] and stillbirth [5] are more likely following ART, even among singletons. These increased risks may be due to one or more elements of ART treatment, the underlying subfertility, other pre-existing medical conditions or a combination of these factors [6].

Comparing the outcomes of ART-conceived births to non-ART births cannot separate the effects of treatment and subfertility; however, it has been difficult to identify more appropriate comparison groups. Births conceived using ovulation induction alone (to women who are also subfertile) are generally not recorded in ART registers, and there is no obvious measure of subfertility in large population-based datasets.

In recent years, some ART outcome studies have included a subfertile comparison group—conceptions arising from parents who previously received subfertility investigation or treatment but subsequently conceived without ART [7–11]. For example, Declercq and colleagues [7] identified a subfertile conception group using a combination of routinely collected data from births, hospital and ART. When compared to ART and fertile groups, the authors reported that the subfertile group was demographically more similar to the ART group than to the fertile group and also had similar rates of pre-existing medical conditions. Births conceived using ovulation induction (OI) and other less invasive fertility treatments often contaminate subfertile comparison groups as these treatments are not recorded on ART registers in most countries. Relatively few studies have reported on outcomes following OI [9, 12, 13] although several large linkage studies have recently accessed prescription dispensing data to identify these births [14, 15].

In general, these studies have tended to show that subfertile treated and untreated women are more similar to each other than to fertile women in terms of demographic characteristics and pre-existing medical conditions. They experience more pregnancy complications and adverse outcomes at delivery [9–11]. When births to subfertile treated and untreated women are compared to each other, births to women who use ART appear to have worse outcomes than births to women who use less invasive treatments and subfertile women who conceive without treatment [6, 9, 11].

In this study, we identify ART, OI, subfertile untreated and fertile natural conceptions using whole-population administrative data. Our main objective with this baseline study is to improve our understanding of underlying differences in demographic data, pre-existing medical conditions and adverse obstetric history across a range of comparison groups identified for ART health outcomes research. Our secondary objective is to examine whether excess risks of pregnancy complications and adverse birth outcomes in ART births remain after comparison with subfertile (untreated and OI) conception groups.

## Methods

### Data sources and linkage

We accessed unit-record, linked data from the following datasets, all of which collect whole-population data by statutory requirement: (i) the Midwives’ Notification System (Midwives’ data)—antenatal and perinatal data on all births ≥ 20 weeks gestation (or ≥ 400 g if age is unknown) in Western Australia (WA); (ii) Hospital Morbidity Data Collection (hospital data)—clinical information about all in-patient hospital admissions including day procedures at public and private hospitals in WA; (iii) the WA Registry of Births, Deaths and Marriages (births and deaths data) iv) Reproductive Technology Register (ART data)—all ART treatment cycles undertaken at fertility clinics in WA; (v) Commonwealth Pharmaceutical Benefits Scheme (PBS) claims (pharmacy data)—an Australian Government programme that subsidises the cost of medicines for most medical conditions. The dataset contains a record of all PBS-listed medications dispensed in the community, private and public hospitals.

Strict legislative and policy barriers prohibit the release of identifiers from WA Health datasets for linkage with Commonwealth data. Pharmacy data were instead linked to the WA Registrar General’s birth and death data by the Australian Institute of Health and Welfare and supplied by the Department of Human Services, and remaining datasets were linked within WA by the WA Data Linkage Branch (WADLB), Australia’s longest running data linkage agency. The WA Data Linkage System is considered one of the most comprehensive, high-quality linkage systems worldwide employing numerous automated and manual sub-processes to reduce the likelihood of linkage error [16–18]. Probabilistic matching was used by both linkage agencies, and data were deidentified for provision to researchers.

### Selection of cohort

The cohort comprised all live births and stillbirths recorded in the WA Midwives’ data during the study period (1 April 2003, to 31 December 2014). We excluded births to Aboriginal women (*N* = 20,324 deliveries) as these comprised a small proportion of the ART group (0.5%) compared with the fertile naturally conceived group (6.5%) and were at higher risk of poor perinatal outcomes [19]. We also excluded 2756 deliveries with an indicator of fertility treatment on the Midwives’ record but no link to an ART treatment cycle or OI medication dispensing. (The treatment flag includes fertility drugs, any ART treatment, any use of donor, intrauterine insemination or tubal transfer [20]). These births were excluded as our goal was to identify subfertile women who we could be sure had used either ART, OI or no treatment at all for comparison with our fertile natural conception group. The final study sample comprised 333,561 births (328,740 deliveries): 323,986 singletons, 9363 twins and 212 higher-order multiples.

### Identifying conception groups

**Fig. 1:**
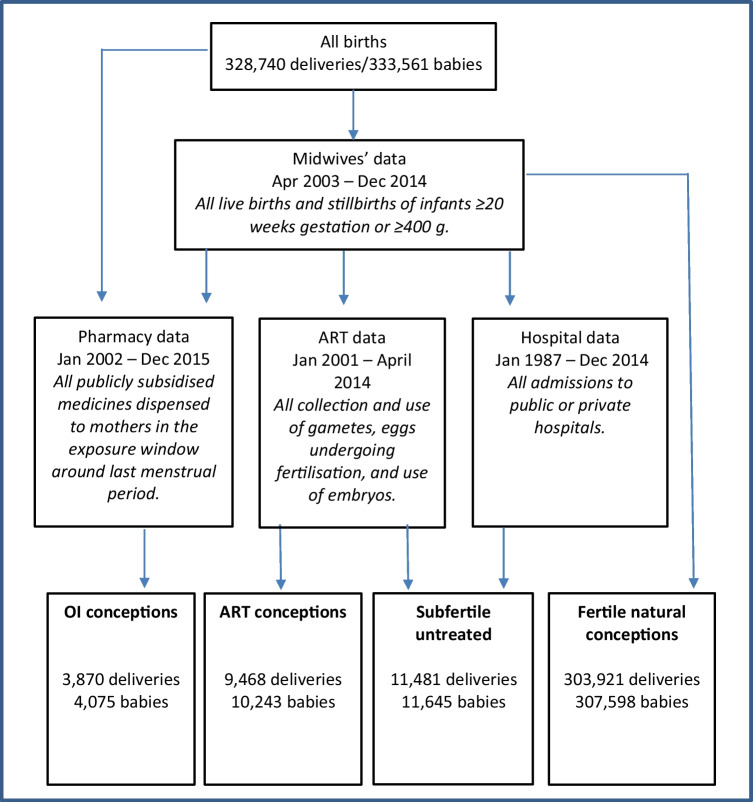
Data sources used to identify conception groups. Ovulation induction (OI) conceptions are those with OI medication dispensed within the relevant time period prior to last menstrual period (LMP). ART conceptions are those that linked to an ART cycle on the Reproductive Technology Register (RTR). Subfertile untreated conceptions are deliveries with any of the following in the 5 years prior to birth—ART cycle on RTR, fertility treatment flag associated with a *previous* birth in Midwives’ data, infertility diagnosis in hospital data. Fertile natural conceptions represent all other births

The last menstrual period (LMP) was defined as date of birth minus gestational age in weeks as provided in the Midwives’ data. To account for potential errors in the estimated gestational age, we allowed a window of ± 3 weeks around estimated LMP. For all births ≥ 20 weeks gestation, we identified four conception groups: (i) ART, (ii) ovulation induction, (iii) subfertile untreated and (iv) fertile naturally conceiving (Fig. 1). The *ART* conception group comprised all births occurring within 120–294 days after a maternal ART cycle listed on the Reproductive Technology Register. This group included women receiving either standard IVF or ICSI treatment (whether fresh or frozen-thawed embryos were transferred).

The *ovulation induction* (OI) conception group was identified by the dispensing of clomiphene, human chorionic gonadotropin, letrozole or tamoxifen within selected time periods prior to the LMP (see Supplemental Table 1). Women dispensed letrozole or tamoxifen were excluded from the OI group if they had a diagnosis of breast cancer in hospital data in the 15 years prior to each birth. These therapies were selected because they were the most commonly used medicines for OI in WA during the study period and were captured in the pharmacy data. The time period between medicine dispensing and LMP differed for each medicine based on the dose supplied, pack size supplied and typical protocols for ovulation induction. From this, we calculated the maximum number of menstrual cycles each dispensing would reasonably last (see Supplemental Table 1). We did not identify dispensing of follitropin alfa or beta because response and doses administered vary widely between individuals, making it difficult to determine how many cycles a dispensing could last. Given that follitropin alfa and beta are always followed by a human chorionic gonadotropin trigger injection, we identified the latter. Births associated with ovulation induction and insemination (donor or intrauterine) could not be distinguished from ovulation induction alone and were classified as ovulation induction conceptions (OI).

Before April 2012, the PBS dataset did not capture dispensing to general beneficiaries (those not receiving social security) which were priced below the patient co-payment (A$23.10 in 2003) [21]. Clomiphene was priced above the co-payment, and therefore captured in the dataset, throughout the study period except for some brands between January and June 2009 and again from January to June 2012. From July 2012, all dispensing was reliably captured irrespective of price. Almost all of our WA maternal cohort (women giving birth from 2003 to 2014) was linked to Commonwealth prescription data (linkage rate, 98.6%), and we assumed that the remaining 1.4% women were not exposed to OI medications.

The *subfertile untreated group* comprised women with a history of subfertility but no evidence of ART or OI in the above-defined periods prior to birth. We defined the history of subfertility, based on an algorithm proposed by Declercq and colleagues [7] modified for the Western Australian context, as any of the following in the 5-year period prior to birth: any procedure in the ART register, fertility treatment indicator associated with a *previous* birth in Midwives’ data or diagnosis of infertility in hospital data (see Supplemental Table 2 and 3). All remaining births were classified as *fertile naturally conceived (fertile).*

### Characteristics of interest

The characteristics we compared among the four conception groups are shown in Table 2. Parental characteristics were identified from hospital inpatient and Midwives’ data and the Registrar General’s file of births. Maternal pre-existing medical conditions and adverse obstetric history were identified through a combination of Midwives’ and hospital data (primary or additional diagnosis or procedure codes listed for a hospital admission in the 15 years prior to each baby date of birth, using ICD-10-AM, ICD-9-CM and Medicare Benefit Schedule codes [22]) (Supplemental Table 2 and 3). Additional maternal pre-existing medical conditions were identified using the Rx-Risk comorbidity index [23] based on pharmacy claims which were available for a minimum of 1 year and a maximum of 12 years prior to each baby date of birth (Supplemental Table 4). BMI was calculated for women giving birth from 2012 to 2014 when both height and weight were recorded in Midwives’ data. Complications of pregnancy, labour and delivery were identified by ICD codes listed for a hospital admission between the LMP and date of birth for each baby (Supplemental Table 2) as well as those recorded in the Midwives’ birth record.

### Statistical analysis

Parental characteristics, maternal pre-existing medical conditions, adverse obstetric history and pregnancy complications were compared across conception groups. Categorical variables were compared using chi-square tests and continuous variables with one-way analysis of variance. Given the large sample size, we discuss differences in prevalence rather than focussing on *P*-values.

We estimated risk ratios (RRs) and 95% confidence intervals (CI) for a range of pregnancy complications and perinatal outcomes in singleton births across conception groups using generalised estimating equations with a Poisson link function and exchangeable correlation structure. Fertile and subfertile untreated conceptions were considered the reference group in separate analyses. Year of birth group (2003–2006, 2007–2010, 2011–2014), maternal age (≤ 28, 29–34, 35 +), parity (primiparous, multiparous) and smoking during pregnancy (yes/no) were included in all models. For the remaining covariates, backward elimination was used to arrive at the final model for each outcome: maternal socioeconomic status (SES) group (area-based social disadvantage index in centiles) [24], private health insurance (PHI) at birth (yes, no), maternal ethnic origin (Caucasian, non-Caucasian), marital status (married and/or cohabiting, other), child’s sex, pre-existing medical conditions (essential hypertension, diabetes, epilepsy, thyroid disorders, endometriosis, fibroid diagnosis or excision, polycystic ovarian syndrome (PCOS), Crohn’s disease or ulcerative colitis, asthma, anxiety or depression and previous cervical biopsy/destruction of lesion) and adverse obstetric history (prior abortion, uterine curettage, ectopic pregnancy, prior preterm birth, prior stillbirth, prior caesarean). The presence of a vanishing twin in the current pregnancy was included in analyses of preterm birth, low birth weight and perinatal death. Vanishing twins were defined as all cases where there was prior death of a co-twin or higher-order sibling before birth, regardless of gestational age. We did not adjust for paternal age (highly correlated with maternal age) or BMI (unable to be calculated for births 2003–2011). There was very little missing data apart from BMI (private health insurance status at birth, 1.1%; marital status, 0.8%; SES, 0.1%), and list-wise deletion from models was considered appropriate given the large study size. Supplementary analyses were used to investigate the impact of missing data on RR estimates of preterm birth, low birth weight and perinatal death. Data were analysed with SPSS version 29 (IBM SPSS Statistics, IBM Corporation, Armonk, NY).

### Ethics approval

Ethics approval for this project was obtained from the WA Department of Health HREC (#2015_65), the WA Reproductive Technology Council and the Australian Institute of Health and Welfare (EO2015/4/211).

## Results

There were 328,731 deliveries of known conception type ≥ 20 weeks gestation during the study period: 9456 (2.9%) were ART conceptions; 3870 (1.2%) were OI conceptions; 11,484 (3.5%) were subfertile untreated conceptions, and 303,921 (92.4%) were fertile conceptions. The prevalence of multiple births was 8% in ART deliveries, 5.1% in OI deliveries and 1.4% and 1.2% in subfertile and fertile conceptions, respectively.

Parental characteristics associated with deliveries in the four conception groups are shown in Table 1. Couples using ART were 5 years older than couples in the fertile group, while subfertile untreated and OI mothers and fathers were 3 and 1 year older, respectively. ART mothers were more often primiparous, least likely to smoke during pregnancy and most likely to have private health insurance and to be of higher SES. The subfertile untreated and OI conceptions were more similar to the ART group than the fertile conceptions in most respects, although subfertile untreated mothers were much more likely to have had previous children (67% vs. 37% of ART women) and women using OI were younger and more likely to deliver a multiple birth than the subfertile untreated women. The broader group of subfertile women (ART, OI and subfertile untreated) were more likely to be married or cohabiting and to have Caucasian ethnicity than fertile women. ART deliveries represented an increasing proportion of the cohort over time (2.0% of births from 2003 to 2006 and 3.4% of births from 2011 to 2014), whereas OI deliveries decreased over the same period (from 1.5 to 0.9% of births).

**Table 1:** Parental characteristics, chronic medical conditions and previous adverse obstetric history in the four conception groups^a^

	Fertile	Subfertile untreated	Ovulation induction	ART^b^	*P*-value
*N* deliveries	303,921 (92.5%)	11,481 (3.5%)	3870 (1.2%)	9468 (2.9%)	
Parental characteristics					
Mother’s age at delivery^c^ Median	29.6 (5.5) 30	33.1 (4.8) 33	31.3 (4.6) 31	34.7 (4.4) 35	< 0.001
Father’s age at delivery^c^ Median	32.4 (6.4) 32	35.5 (5.7) 35	33.8 (5.4) 33	37.7 (6.2) 37	< 0.001
Marital status					
Married or cohabiting Other Missing	269,188 (88.6%) 32,196 (10.6%) 2537 (0.8%)	10,935 (95.2%) 485 (4.2%) 61 (0.5%)	3742 (96.7%) 112 (2.9%) 16 (0.4%)	9071 (95.8%) 349 (3.7%) 48 (0.5%)	< 0.001
Maternal ethnic origin					
Caucasian Other	248,925 (81.9%) 54,996 (18.1%)	10,219 (89.0%) 1262 (11.0%)	3495 (90.3%) 375 (9.7%)	8356 (88.3%) 1112 (11.7%)	< 0.001
SES percentile group					
< 10% 10 ≤ 24% 25 ≤ 75% 75 ≤ 90% ≥ 90% Missing	21,666 (7.1%) 46,183 (15.2%) 150,125 (49.4%) 49,023 (16.1%) 36,602 (12.0%) 322 (0.1%)	680 (5.9%) 1451 (12.6%) 5463 (47.6%) 1957 (17.0%) 1928 (16.8%) < 5	235 (6.1%) 504 (13.0%) 1913 (49.4%) 615 (15.9%) 599 (15.5%) < 5	389 (4.1%) 931 (9.8%) 4464 (47.1%) 1781 (18.8%) 1902 (20.1%) < 5	< 0.001
Private health insurance					
Yes No Missing	110,011 (36.2%) 190,526 (62.7%) 3384 (1.1%)	7373 (64.2%) 4035 (35.1%) 73 (0.6%)	2593 (67.0%) 1256 (32.5%) 21 (0.5%)	7265 (76.7%) 2140 (22.6%) 63 (0.7%)	< 0.001
Smoked during pregnancy	38,013 (12.5%)	773 (6.7%)	186 (4.8%)	244 (2.6%)	< 0.001
Parity					
0 1 2 ≥ 3	129,277 (42.5%) 104,222 (34.3%) 45,727 (15.0%) 24,695 (8.1%)	3751 (32.7%) 5218 (45.4%) 1842 (16.0%) 670 (5.8%)	2076 (53.6%) 1299 (33.6%) 365 (9.4%) 130 (3.4%)	5915 (62.5%) 2762 (29.2%) 539 (5.7%) 252 (2.7%)	< 0.001
Year of baby’s birth					
2003–2006 2007–2010 2011–2014	84,445 (27.8%) 104,132 (34.3%) 115,344 (38.0%)	3033 (26.4%) 4072 (35.5%) 4376 (38.1%)	1403 (36.3%) 1309 (33.8%) 1158 (29.9%)	1845 (19.5%) 3379 (35.7%) 4244 (44.8%)	< 0.001
Plurality					
Singleton Multiple	300,285 (98.8%) 3636 (1.2%)	11,319 (98.6%) 162 (1.4%)	3671 (94.9%) 199 (5.1%)	8711 (92.0%) 757 (8.0%)	< 0.001
Maternal chronic conditions					
Anxiety or depression^d,e^	53,249 (17.5%)	2284 (19.9%)	669 (17.3%)	1423 (15.0%)	< 0.001
Asthma^e,f^	36,227 (11.9%)	1436 (12.5%)	536 (13.9%)	1039 (11.0%)	< 0.001
Crohn’s disease or ulcerative colitis^e^	1307 (0.4%)	74 (0.6%)	30 (0.8%)	80 (0.8%)	< 0.001
Diabetes^e,f^	2143 (0.7%)	162 (1.4%)	50 (1.3%)	113 (1.2%)	< 0.001
Epilepsy^d,e^	4254 (1.4%)	177 (1.5%)	56 (1.4%)	101 (1.1%)	0.025
Hypertension^e,f^	3774 (1.2%)	206 (1.8%)	73 (1.9%)	184 (1.9%)	< 0.001
BMI^f^ (for women 18 + , with births 2012–2014)					
Underweight Healthy weight Overweight Obese Class 1 obese Class 2 obese Class 3 obese Missing	2120 (2.6%) 39,266 (47.9%) 23,359 (28.5%) 17,175 (20.9%) 10,738 (13.1%) 4273 (5.2%) 2164 (2.6%) 5460 (6.2%)	70 (2.2%) 1519 (48.3%) 912 (29.0%) 641 (20.4%) 419 (13.3%) 172 (5.5%) 50 (1.6%) 221 (6.6%)	23 (2.9%) 357 (45.5%) 192 (24.5%) 213 (27.2%) 135 (17.2%) 43 (5.5%) 35 (4.5%) 64 (7.5%)	80 (2.6%) 1597 (51.3%) 888 (28.5%) 551 (17.7%) 425 (13.6%) 107 (3.4%) 19 (0.6%) 192 (5.8%)	< 0.001
PCOS^e^	735 (0.2%)	574 (5.0%)	167 (4.3%)	472 (5.0%)	< 0.001
Endometriosis^e^	4405 (1.4%)	3031 (26.4%)	397 (10.3%)	2188 (23.1%)	< 0.001
Fibroids—diagnosis or excision^e^	1660 (0.5%)	619 (5.4%)	81 (2.1%)	573 (6.1%)	< 0.001
Thyroid disorder^d,e^	4514 (1.5%)	310 (2.7%)	103 (2.7%)	299 (3.2%)	< 0.001
Destruction of lesion or biopsy cervix^e^	6509 (2.1%)	544 (4.7%)	139 (3.6%)	368 (3.9%)	< 0.001
Previous adverse obstetric history					
Abortion^e^ (spontaneous or medical)	58,788 (19.3%)	3223 (28.1%)	911 (23.5%)	1999 (21.1%)	< 0.001
Uterine Curettage^e^	39,044 (12.8%)	4807 (41.9%)	1067 (27.6%)	3309 (34.9%)	< 0.001
Ectopic pregnancy^e^	3162 (1.0%)	449 (3.9%)	113 (2.9%)	571 (6.0%)	< 0.001
* N* (parity > 0)	174,644	7730	1794	3553	
Preterm birth^e,f,g^	9449 (5.4%)	678 (8.8%)	134 (7.5%)	363 (10.2%)	< 0.001
Stillbirth^e,f,g^	3638 (2.1%)	243 (3.1%)	66 (3.7%)	160 (4.5%)	< 0.001
Caesarean section^f,g^	49,465 (28.3%)	3246 (42.0%)	658 (36.7%)	1510 (42.5%)	< 0.001

^a^Denominator = deliveries

^b^Assisted Reproductive Technology

^c^Mean, standard deviation

^d^Determined from pharmacy claims

^e^Determined from hospital records

^f^Determined from midwives’ birth records. Where more than one superscript, determined from combined results of more than one dataset

^g^Restricted to women with parity > 0

### Pre-existing maternal chronic conditions

Mothers using ART were less likely to have asthma, epilepsy or a history of medication use for anxiety or depression than the other groups of women (Table 1). For birth years 2012–2014 (where BMI could be calculated), mothers using ART were least likely to be obese. The broader group of subfertile women (ART, OI and subfertile untreated) was more likely to have pre-existing diabetes, hypertension, a thyroid disorder as well as indicators of Crohn’s disease or ulcerative colitis in hospital data than fertile mothers. They were also much more likely to have a diagnosis of PCOS (4.3–5% vs. 0.2% of fertile women). Diagnosis or excision of fibroids and endometriosis were most common in ART and subfertile untreated women, while those using OI had proportionately less of these diagnoses although still more than fertile women.

### Adverse obstetric history

Subfertile untreated women were the most likely to have a history of prior abortion (spontaneous or medical) followed by women who had used OI. Less than 13% of fertile women had a history of prior curettage compared with 28% of women using OI, 35% of women using ART and 42% of subfertile untreated women. Prior ectopic pregnancy was most frequently reported for women using ART (6.0% vs. 1–3.9%). When we restricted analyses to women with previous births, all subfertile groups were more likely to have a prior preterm birth, prior stillbirth and prior caesarean section with the greatest risks seen in women using ART.

### Complications of pregnancy, labour and delivery

Table 2 shows the prevalence of complications of pregnancy, labour and delivery observed for singleton babies ≥ 20 weeks gestation in the four conception groups (data for multiple births are included in Supplemental Table 5). Some complications were increased across all three groups of subfertile births (ART, OI and subfertile untreated), including cervical incompetence, gestational diabetes and threatened preterm labour (< 37 weeks). Babies born to subfertile treated women (OI and ART) were more likely to experience preeclampsia than the subfertile untreated and fertile conception groups.

**Table 2:** Complications of pregnancy, labour and delivery for singleton births across conception groups^a^

	Fertile	Subfertile untreated	Ovulation induction	ART^b^	*P*-value
Singleton births, *N*	300,285	11,319	3671	8711	
Anaemia^e^	7345 (2.4%)	180 (1.6%)	74 (2.0%)	191 (2.2%)	< 0.001
Cervical incompetence^e^	796 (0.3%)	155 (1.4%)	42 (1.1%)	99 (1.1%)	< 0.001
Urinary tract infection^e,f^	11,115 (3.7%)	385 (3.4%)	128 (3.5%)	249 (2.9%)	< 0.001
Infection of amniotic sac and membranes^e^	947 (0.3%)	40 (0.4%)	16 (0.4%)	46 (0.5%)	0.003
Vanishing twin^e^	95 (0.0%)	5 (0.0%)	< 5	29 (0.3%)	< 0.001
Preeclampsia/eclampsia^e,f^	11,807 (3.9%)	393 (3.5%)	193 (5.3%)	464 (5.3%)	< 0.001
Gestational hypertension without proteinuria^e^	15,599 (5.2%)	535 (4.7%)	238 (6.5%)	492 (5.6%)	< 0.001
Gestational diabetes^e,f^	20,047 (6.7%)	1005 (8.9%)	340 (9.3%)	910 (10.4%)	< 0.001
Morbidly adherent placenta^e^	1113 (0.4%)	69 (0.6%)	22 (0.6%)	86 (1.0%)	< 0.001
Placenta praevia^f^	1382 (0.5%)	90 (0.8%)	27 (0.7%)	153 (1.8%)	< 0.001
Placental abruption^e,f^	1976 (0.7%)	74 (0.7%)	27 (0.7%)	89 (1.0%)	< 0.001
Threatened abortion < 20 weeks^e,f^	9973 (3.3%)	720 (6.4%)	229 (6.2%)	801 (9.2%)	< 0.001
Other antepartum haemorrhage^e,f^	12,194 (4.1%)	552 (4.9%)	178 (4.8%)	609 (7.0%)	< 0.001
Poor foetal growth^e^	9648 (3.2%)	339 (3.0%)	128 (3.5%)	351 (4.0%)	< 0.001
Excessive foetal growth^e^	8229 (2.7%)	368 (3.3%)	128 (3.5%)	315 (3.6%)	< 0.001
Threatened preterm labour < 37 weeks^e,f^	12,353 (4.1%)	675 (6.0%)	196 (5.3%)	466 (5.3%)	< 0.001
Prelabour rupture of membranes^e,f^	26,156 (8.7%)	829 (7.3%)	279 (7.6%)	770 (8.8%)	< 0.001
Complications of labour and delivery					
Vasa praevia^e^	119 (0.0%)	< 5	< 5	15 (0.2%)	< 0.001
Prolapsed cord^f^	338 (0.1%)	20 (0.2%)	< 5	13 (0.1%)	0.190
Shoulder dystocia^e,f^ (vaginal deliveries only)	6798 (3.3%)	196 (3.2%)	52 (2.4%)	131 (3.1%)	0.091
Emergency caesarean^f^	44,573 (14.8%)	1727 (15.3%)	637 (17.4%)	1836 (21.1%)	< 0.001
Postpartum haemorrhage (PPH)^e,f^	47,921 (16.0%)	1463 (12.9%)	467 (12.7%)	1359 (15.6%)	< 0.001
PPH following Vaginal delivery PPH following Elective caesarean PPH following Emergency caesarean	26,900 (13.2%) 8244 (15.9%) 12,777 (28.7%)	694 (11.3%) 392 (11.4%) 377 (21.8%)	260 (12.1%) 80 (9.1%) 127 (19.9%)	612 (14.7%) 340 (12.6%) 407 (22.2%)	< 0.001 < 0.001 < 0.001
Preterm birth—live births only^f^					
< 32 weeks 32–36 weeks	2153 (0.7%) 15,656 (5.2%)	117 (1.0%) 761 (6.8%)	47 (1.3%) 244 (6.7%)	140 (1.6%) 775 (9.0%)	< 0.001
Low birth weight—live births only^f^					
< 1500 g 1500 g to < 2500 g	1849 (0.6%) 10,832 (3.6%)	91 (0.8%) 476 (4.2%)	40 (1.1% 142 (3.9%)	120 (1.4%) 449 (5.2%)	< 0.001
Perinatal death^a,f,g,h^	2284 (0.8%)	103 (0.9%)	26 (0.7%)	103 (1.2%)	< 0.001
Stillbirth	1859 (0.6%)	82 (0.7%)	19 (0.5%)	85 (1.0%)	< 0.001

^a^Denominator = babies

^b^Assisted Reproductive Technology

^d^Determined from pharmacy claims

^e^Determined from hospital records

^f^Determined from Midwives’ records

^g^Determined from death records

^h^Perinatal death refers to a death between 20 weeks gestation and 28 days following birth

Where more than one superscript, determined from combined results of more than one dataset

Other complications were more common in ART pregnancies. These included placental problems (morbidly adherent placenta, placenta praevia and placental abruption), threatened abortion at < 20 weeks, other antepartum haemorrhage, a vanishing twin gestation and delivery by emergency caesarean section. ART singletons were also most likely to be born preterm, low birth weight and to die in the perinatal period (i.e. from 20 weeks gestation to the first 28 days post-birth).

Some patterns were similar in multiple births (see Supplemental Table 5). ART-conceived multiples were again most likely to experience placental abruption, threatened abortion at < 20 weeks and other antepartum haemorrhage; however, placenta praevia was increased across all subfertile groups (ART, OI and subfertile untreated). Multiples conceived using OI experienced more preeclampsia and gestational hypertension. ART multiples were more likely to be born preterm and low birth weight and to be delivered by emergency caesarean section.

### Adjusted analyses for pregnancy complications and perinatal outcomes in singleton births

In crude analyses, all subfertile groups (ART, OI and subfertile untreated) had significantly increased risks of pregnancy complications and adverse perinatal outcomes compared with fertile conceptions, with the exception of preeclampsia (no increase in subfertile untreated), placental abruption and perinatal death (no increase in subfertile untreated or OI conceptions) (Table 3). ART conceptions generally had the greatest risk of complications and adverse outcomes, although they had a similar risk of preeclampsia and a lower risk of gestational diabetes mellitus (GDM) than OI conceptions in adjusted analyses. When analyses were restricted to subfertile women (ART, OI and subfertile untreated), ART conceptions had significantly increased risks of all outcomes compared with subfertile untreated conceptions except for GDM where the risk for these two groups was similar. In contrast, OI conceptions were only at increased risk of preeclampsia and GDM compared with subfertile untreated conceptions. Of note, after adjustment for potential confounders, placenta praevia was still increased 2.4-fold in ART vs. subfertile untreated births, morbidly adherent placenta 1.6-fold, placental abruption 1.8-fold, preterm birth 1.4-fold, low birth weight 1.3-fold and perinatal death 1.4-fold (Table 3). Missing data had minimal impact on the RRs in supplementary analyses (see Supplemental Table 6).

**Table 3:** Crude and adjusted risk ratios for comparison of pregnancy complications and perinatal outcomes across conception groups in singleton births

	Conception group	*N* (%)	Fertile reference		Subfertile reference	
			Crude RR (95% CI)	Adjusted RR (95% CI)	Crude RR (95% CI)	Adjusted RR (95% CI)
Threatened miscarriage < 20 weeks^a^	Fertile	9973 (3.3%)	1.00 (reference)	1.00 (reference)		
	Subfertile untreated	720 (6.4%)	1.92 (1.78–2.06)	1.29 (1.19–1.40)	1.00 (reference)	1.00 (reference)
	OI	229 (6.2%)	1.88 (1.65–2.13)	1.24 (1.09–1.41)	0.98 (0.85–1.13)	0.98 (0.85–1.14)
	ART	801 (9.2%)	2.77 (2.58–2.97)	1.85 (1.71–2.00)	1.44 (1.31–1.59)	1.40 (1.26–1.56))
Morbidly adherent placenta^b^	Fertile	1113 (0.4%)	1.00 (reference)	1.00 (reference)		
	Subfertile untreated	69 (0.6%)	1.64 (1.29–2.10)	1.09 (0.83–1.42)	1.00 (reference)	1.00 (reference)
	OI	22 (0.6%)	1.62 (1.06–2.46)	1.37 (0.90–2.09)	0.98 (0.61–1.59)	1.16 (0.71–1.88)
	ART	86 (1.0%)	2.66 (2.14–3.31)	1.70 (1.34–2.15)	1.62 (1.18–2.22)	1.55 (1.12–2.16)
Placenta praevia^c^	Fertile	1382 (0.5%)	1.00 (reference)	1.00 (reference)		
	Subfertile untreated	90 (0.8%)	1.73 (1.40,2.14)	1.22 (0.97–1.54)	1.00 (reference)	1.00 (reference)
	OI	27 (0.7%)	1.60 (1.09,2.34)	1.32 (0.91–1.93)	0.92 (0.60–1.42)	0.96 (0.62–1.51)
	ART	153 (1.8%)	3.82 (3.23, 4.50)	2.92 (2.41–3.53)	2.21 (1.70–2.86)	2.42 (1.82–3.20)
Placental abruption^d^	Fertile	1976 (0.7%)	1.00 (reference)	1.00 (reference)		
	Subfertile untreated	74 (0.7%)	0.99 (0.79–1.25)	0.87 (0.68–1.13)	1.00 (reference)	1.00 (reference)
	OI	27 (0.7%)	1.12 (0.77–1.63)	1.12 (0.76–1.64)	1.12 (0.72–1.75)	1.19 (0.75–1.87)
	ART	89 (1.0%)	1.55 (1.26–1.92)	1.52 (1.21–1.91)	1.56 (1.15–2.13)	1.78 (1.26–2.50)
Pre-eclampsia^e^	Fertile	11,807 (3.9%)	1.00 (reference)	1.00 (reference)		
	Subfertile untreated	393 (3.5%)	0.88 (0.80–0.98)	0.90 (0.81–1.00)	1.00 (reference)	1.00 (reference)
	OI	193 (5.3%)	1.34 (1.16–1.54)	1.10 (0.96–1.26)	1.51 (1.28–1.79)	1.23 (1.04–1.46)
	ART	464 (5.3%)	1.36 (1.24–1.48)	1.12 (1.02–1.23)	1.53 (1.34–1.75)	1.27 (1.11–1.47)
Gestational diabetes^f^	Fertile	20,047 (6.7%)	1.00 (reference)	1.00 (reference)		
	Subfertile untreated	1005 (8.9%)	1.33 (1.25–1.41)	1.12 (1.05–1.19)	1.00 (reference)	1.00 (reference)
	OI	340 (9.3%)	1.39 (1.25–1.54)	1.34 (1.21–1.49)	1.04 (0.93–1.17)	1.12 (1.00–1.26)
	ART	910 (10.4%)	1.56 (1.47–1.67)	1.16 (1.08–1.24)	1.18 (1.08–1.28)	1.05 (0.96–1.15)
Preterm birth^g,h^	Fertile	17,809 (6.0%)	1.00 (reference)	1.00 (reference)		
	Subfertile untreated	878 (7.8%)	1.31 (1.23–1.40)	1.16 (1.08–1.24)	1.00 (reference)	1.00 (reference)
	OI	291 (8.0%)	1.34 (1.20–1.49)	1.26 (1.12–1.41)	1.02 (0.90–1.16)	1.07 (0.94–1.22)
	ART	915 (10.6%)	1.78 (1.67–1.89)	1.55 (1.45–1.66)	1.36 (1.24–1.48)	1.38 (1.25–1.52)
Low birth weight^g,i^	Fertile	12,961 (4.3%)	1.00 (reference)	1.00 (reference)		
	Subfertile untreated	574 (5.1%)	1.18 (1.08–1.28)	1.16 (1.06–1.26)	1.00 (reference)	1.00 (reference)
	OI	186 (5.1%)	1.17 (1.02–1.35)	1.22 (1.06–1.41)	1.00 (0.85–1.17)	1.02 (0.86–1.20)
	ART	576 (6.7%)	1.54 (1.42–1.67)	1.46 (1.34–1.60)	1.31 (1.17–1.46)	1.26 (1.11–1.42)
Perinatal death^j,k^	Fertile	2284 (0.8%)	1.00 (reference)	1.00 (reference)		
	Subfertile untreated	103 (0.9%)	1.20 (0.98–1.46)	1.26 (1.03–1.54)	1.00 (reference)	1.00 (reference)
	OI	26 (0.7%)	0.93 (0.63–1.37)	1.07 (0.73–1.58)	0.78 (0.51–1.20)	0.85 (0.55–1.31)
	ART	103 (1.2%)	1.56 (1.28–1.89)	1.74 (1.42–2.14)	1.30 (0.99–1.70)	1.37 (1.02–1.84)

Covariates in final adjusted Poisson generalised estimating equation models

^a^Threatened abortion: baby year of birth group, maternal age group, smoking, parity group, marital status, private health insurance at birth, ethnicity (Caucasian vs. other), SES, pre-existing diabetes, anxiety or depression, asthma, epilepsy, PCOS, endometriosis, fibroids, prior abortion, prior ectopic pregnancy, prior uterine curettage, prior stillbirth

^b^Morbidly adherent placenta: baby year of birth group, maternal age group, parity, smoking, sex, epilepsy, thyroid disorder, endometriosis, fibroids, prior preterm birth, prior abortion, prior curettage, prior caesarean

^c^Placenta praevia: baby year of birth group, maternal age group, parity, private health insurance at birth, ethnicity, smoking, asthma, thyroid disorder, endometriosis, prior abortion, prior caesarean

^d^Placental abruption: baby year of birth group, maternal age group, parity, private health insurance at birth, ethnicity, smoking, SES, preexisting hypertension, anxiety or depression, thyroid disorder, endometriosis, fibroids, prior preterm birth, prior curettage

^e^Preeclampsia: baby year of birth group, maternal age group, parity, private health insurance at birth, ethnicity, smoking, SES, preexisting diabetes, preexisting hypertension, anxiety or depression, asthma, epilepsy, thyroid disorder, prior preterm birth, prior stillbirth, prior caesarean

^f^Gestational diabetes: baby year of birth group, maternal age group, parity, private health insurance at birth, marital status, ethnicity, smoking, sex, SES, preexisting diabetes, preexisting hypertension, anxiety or depression, asthma, thyroid disorder, PCOS, fibroids, prior preterm birth, prior abortion, prior curettage, prior stillbirth, prior caesarean

^g^Denominator = livebirths

^h^Preterm birth: baby year of birth group, maternal age group, parity, marital status, private health insurance at birth, ethnicity, smoking, sex, SES, preexisting diabetes, preexisting hypertension, anxiety or depression, asthma, Crohn’s disease or ulcerative colitis, epilepsy, thyroid disorder, fibroids, cervix procedure, vanishing twin survivor, prior preterm birth, prior abortion, prior curettage, prior stillbirth, prior ectopic, prior caesarean

^i^Low birth weight: baby year of birth group, maternal age group, parity, marital status, ethnicity, private health insurance at birth, smoking, sex, SES, preexisting diabetes, preexisting hypertension, anxiety or depression, asthma, Crohn’s disease or ulcerative colitis, epilepsy, thyroid disorder, endometriosis, fibroids, cervix procedure, vanishing twin survivor, prior preterm birth, prior curettage, prior stillbirth, prior ectopic, prior caesarean

^j^Denominator = livebirths and stillbirths

^k^Perinatal death: baby year of birth group, maternal age group, parity, marital status, private health insurance at birth, ethnicity, smoking, SES, preexisting diabetes, preexisting hypertension, Crohn’s disease or ulcerative colitis, fibroids, vanishing twin survivor, prior preterm birth, prior curettage, prior stillbirth

## Discussion

Our results confirm the previously identified increased risks of pregnancy complications and adverse birth outcomes in subfertile women [9, 11, 25]. These women had more pre-existing chronic conditions and were more likely to have an adverse obstetric history than fertile women. Our goal was to examine how similar our new subfertile untreated and OI conception groups were to ART births in terms of demographics, pre-existing medical conditions, adverse obstetric history and complications of pregnancy and delivery before using them as comparison groups in further ART research. Generally, we found that our new comparison groups were more similar to the ART group than to fertile conceptions; however, some important differences remained. The proportion of parous women was much higher in the subfertile untreated group. Women who used OI were younger and more obese than the subfertile untreated and ART groups; they were also less likely to have endometriosis or fibroids (although still much more likely to have these conditions than fertile women). Women using ART were healthier in terms of reduced obesity and smoking, but they were more likely to have a prior ectopic pregnancy, prior preterm birth, and prior stillbirth compared with other subfertile groups.

When we examined several pregnancy complications (threatened abortion, placental problems, preeclampsia, gestational diabetes) and birth outcomes in adjusted analyses, we found that ART-conceived singleton births had greater risks of pregnancy complications and adverse outcomes than births to fertile women although absolute risks remained small. Risks were also increased compared to subfertile untreated women for all outcomes except GDM. In contrast, singleton births conceived using OI generally had a similar risk profile to subfertile untreated births, although they were at greater risk of preeclampsia and GDM. Women with PCOS are more likely to undertake OI as a first-line fertility treatment for anovulation, and the hormonal/metabolic derangements associated with this condition have been proposed to increase risks of pre-eclampsia and GDM [26]. Obesity is also known to increase the risk of these pregnancy complications [27], and although we were only able to calculate BMI for births in the last 3 years of the study, we observed more prevalent and more severe obesity in the women using OI.

In agreement with many other studies [4, 11, 28, 29], ART-conceived singleton births were at particular increased risk of placental problems including a > twofold increased risk of placenta praevia compared with OI, subfertile untreated and fertile births, although absolute risks were small (1.8% ART singletons versus 0.7% OI, 0.8% subfertile untreated and 0.5% fertile births). All singleton births to subfertile women (treated and untreated) were at increased risk of low birth weight and preterm birth with the greatest risks apparent for ART births, while only ART and untreated subfertile births were at increased risk of perinatal death. Similar risk patterns across conception groups have been described in the US MOSART cohort and the Canadian study of preterm birth by Wang et al. [9, 10]. For example, Wang et al*.* [9] report almost identical increasing prevalence of preterm birth from unassisted conception (5.9%) to subfertility without treatment (7.7%), OI/IUI treatment (8.1%) and ART (10.8%). Other studies without a subfertile untreated comparison group have reported increased risks of preterm birth and low birth weight for OI births intermediate between natural conceptions and ART births [12, 13, 30, 31]. We were able to account for a greater range of chronic medical conditions and adverse obstetric history in adjusted analyses than previous studies; however, we were unable to adjust for BMI. We did not differentiate between provider-initiated and spontaneous preterm birth, although Wang et al*.* [9] showed consistently higher risks of both types of preterm birth in ART singletons compared with OI, subfertile untreated and fertile conceptions.

Although OI and subfertile untreated conceptions are more similar to ART conceptions, women using ART may still represent a more severely subfertile group of women, some of whom could only ever hope to conceive using ART. This is supported by a much higher proportion of primiparity (62.5%) compared with our subfertile untreated (32.7%), and to a lesser extent OI (53.6%), conception groups. We investigated whether this difference in parity was related to our method for identifying subfertile untreated births, specifically the inclusion of births that had a fertility treatment flag on a *previous* birth in Midwives’ data (dependent on having a prior birth). However, only 4% of our subfertile untreated group were identified solely on this basis—all others had prior diagnoses of subfertility in hospital data or prior ART cycles. While the proportion of women with endometriosis, fibroids and PCOS was very similar in our ART and subfertile untreated group, we were unable to assess the severity of these underlying reproductive disorders. It is likely that we are under-ascertaining these conditions through hospital inpatient data, and the women identified with these disorders probably represent women at the more severe end of the subfertility spectrum. It is difficult to estimate the extent of potential under-ascertainment since reports are often made of the prevalence of these conditions in reproductive age women, rather than women giving birth. Since these conditions are associated with infertility, the prevalence is likely reduced in women giving birth. Endometriosis, for example, is thought to affect 10% of reproductive age women [26] and was ascertained in hospital data for only 3% of deliveries in our cohort and 2.4% of a recent Danish singleton cohort (1989–2013) [32]. PCOS is reported to occur in 7–14% of reproductive age women and was diagnosed in only 0.6% of our cohort compared to 1.3% of a Swedish cohort 2005–2014 [33] where data were also available on outpatient specialist care (both public and private). We expected to see a greater proportion of women with PCOS in our OI group for whom this is generally the first-line treatment; however, OI medications can only be prescribed by authorised practitioners in Australia (generally endocrinologists or obstetricians/gynecologists), and diagnoses of PCOS for these women may never appear in hospital data.

In addition to the potential for more severe subfertility in women undergoing ART, there may be aspects of the treatment itself that further complicate pregnancies in women with reproductive disorders. For example, Stern et al*.* [11] compared ART-treated and untreated women with diagnoses of tubal factor, PCOS, other ovulatory disorder and endometriosis determined through hospital and insurance claims data. They found increased risks for the ART-treated pregnancies and deliveries compared with the untreated groups, including gestational hypertension, placental abnormalities, preterm birth and low birth weight. Sibling studies also suggest small additional risks among ART vs. naturally conceived births to the same mother [34–36]. Differences in risk profile for births following fresh versus frozen embryo transfer (FET) also indicate treatment factors may play a role. Previous studies report that fresh embryo transfer and controlled ovarian hyperstimulation are associated with small for gestational age, placenta praevia, preterm birth and low birth weight [6]. In contrast, FET is associated with large for gestational age and preeclampsia with particular risks related to the use of hormone preparation cycles to prepare the endometrium for frozen embryo replacement [37, 38].

Stern et al*.* [10] propose that abnormal placentation contributes to the greater risks of preterm birth and low birth weight associated with ART treatment. The aetiology of many placental disorders is still unknown, although placenta praevia (increased threefold in ART singleton births in this study) is thought to occur largely because of damage to the endometrium caused by surgeries (prior caesarean delivery) or prior miscarriages. These lead to the formation of scar tissue that may modify the direction of uterine contractility and the flow of endometrial secretions [39]. Some subfertile women may also have an underlying endometrial problem which may be the reason for their infertility. Underlying reproductive disorders like endometriosis and uterine fibroids have also been associated with increased risks of placenta praevia [26]. In agreement with these observations, prior abortion, prior caesarean section and endometriosis were all independently associated with placenta praevia in our study, although they could not explain the excess risk associated with ART. We did not examine risk associated with specific aspects of ART treatment; however, Carusi et al*.* [40] report that the use of controlled ovarian hyperstimulation in the context of fresh embryo transfer was the only identified ART cycle-related factor associated with placenta praevia, increasing risk by 31%. Given the much higher risk of placenta praevia seen in ART births, the authors postulate that other factors such as a difficult transfer or uterine contractions must be involved. Other authors have also suggested that transcervical embryo transfer may itself play a role, possibly stimulating uterine contractions or altering uterine flow [36, 39].

## Strengths and limitations

The strengths of this large study spanning more than a decade include linkage of statutory, whole-population datasets enabling us to identify different methods of conception and to examine rare outcomes such as perinatal death. Small amounts of missing data for three covariates did not appear to materially affect our RR estimates.

We are almost certainly underestimating the number of subfertile women who conceived naturally, since consultation and/or treatment for subfertility may often occur in an outpatient setting or in private practice. We relied on inpatient hospital diagnoses, Midwives’ birth data and prior history of ART treatment to identify our subfertile group, whereas other studies [9, 41] were able to identify larger subfertile groups through the addition of outpatient diagnoses of infertility. Indeed, 16% of couples in Australia are reported to have problems with fertility [42, 43], greater than all our subfertile (treated, untreated and excluded) groups combined (9%). When Stern et al*.* [41] compared a larger subfertile group identified through an insurance claims database with their original subfertile group [7] identified through hospital inpatient, birth and ART data (as in our study), they reported very similar demographic parameters, underlying health conditions, and pregnancy and delivery outcomes, intermediate between those of the ART and fertile groups [41]. We therefore anticipate minimal impact on our study results since the subfertile women misclassified as fertile are likely similar to those we have identified and will form only a very small proportion of our much larger fertile group, having little influence on the results for that group.

We excluded births with an indicator of fertility treatment in their Midwives’ record that did not link to an ART treatment cycle or OI dispensing record to prevent contamination of our subfertile comparison group with treated women. This differs from the majority of US studies using MOSART data where women with indicators of fertility treatment for their current birth that had not linked to an ART cycle were included in the subfertile comparison group. The excluded births in our study likely represent a mix of conception types including donor insemination or IUI treatment without OI (these represented 0.3% of a similar birth cohort described in New South Wales) [14]; OI treatment that we failed to identify through linkage with PBS data due to two 6-month periods where some brands of clomiphene fell below co-payment [15] or where a woman may have used OI medicines that were left over from a previous dispensing/pregnancy attempt, and women who sought fertility treatment overseas (often to access donor oocytes) and returned to WA to give birth [44].

We report a slightly lower prevalence of OI conception (1.2%) than other states in Australia-NSW/ACT (1.9% mixed OI/IUI/DI) [14] and SA (1.6% clomiphene births) [15], but the same prevalence as Wang et al*.* in a large Canadian linkage study covering a similar time frame (2006–2014) [9]. Identification of our OI group is reliant on medication dispensing rather than consumption; however, we consider that OI medication adherence is likely to be high because the women are seeking assistance to achieve a desired goal of pregnancy, they are known to have obtained the drug from the pharmacy, the treatment regime is uncomplicated and medication use is short term. Use of OI medications is associated with an increased risk of multiple pregnancies [12, 13], and we report a multiple pregnancy rate of 5.1%, similar to the 4.9% and 5.7% reported for OI births in NSW/ACT [14] and SA [15], and much higher than the 1.2–1.4% observed in our fertile and subfertile untreated comparison groups, suggesting that these women were indeed exposed to fertility medicines.

Hospital data will generally underestimate the prevalence of most medical conditions. Where possible, we used a combination of Midwives’, hospital and sometimes PBS data to capture pre-existing conditions, adverse obstetric history and pregnancy complications. For example, we combined Midwives’ and hospital data for pre-existing diabetes and reported this condition in 0.75% of deliveries which corresponds well with national Australian data [45, 46]. We did not examine complications of pregnancy and delivery outcomes by medication type in OI births or by different treatment factors in ART births. Treatment-specific effects will be examined in future analyses. Finally, it is important to emphasise that these data relate to births from 2003 to 2014, ART techniques are rapidly changing and there may be fewer pregnancy complications and adverse birth outcomes associated with more recent ART births.

## Summary

Our primary goal was to identify conception groups that could assist in separating ART treatment effects from the effects of subfertility itself. We found that the OI and subfertile untreated groups were more similar to the ART group than to the fertile group and therefore offer improved options for interpreting health outcomes in ART-conceived births. However, despite the closer similarities between the OI and subfertile untreated groups, some differences remained with respect to parity, maternal age, obesity, adverse obstetric history and others. In multivariate analyses, we found that OI singleton births have a risk profile that resembles subfertile untreated births with additional risk of GDM and preeclampsia. In contrast, and despite similar underlying reproductive disorders and adverse obstetric history in ART and untreated subfertile women, ART singleton births carried a greater risk of most pregnancy complications and adverse birth outcomes including preterm birth, low birth weight and perinatal death. Placental disorders were again highlighted as a key risk area for ART births. Disentangling individual pathways, even with additional comparison groups, is very challenging, and it is likely that a range of factors, including underlying reproductive and other disorders, obstetric history and aspects of treatment may act together to dysregulate normal placentation giving rise to the excess risks seen after ART.

### Supplementary Information

Below is the link to the electronic supplementary material.

Supplementary file1 (DOCX 47 KB)

## Data Availability

The data that support the findings of this study are not publicly available due to privacy and ethical restrictions.
