# Supplementary material for: Ovulation induction and subfertile untreated conception groups offer improved options for interpreting risks associated with ART
Source: J Assist Reprod Genet. 2024 Mar 12;41(4):915–28. doi: 10.1007/s10815-024-03060-6 (PMC11052736; doi:10.1007/s10815-024-03060-6)
Supplement: Supplementary file 1 — Supplementary file1 (DOCX 47 KB) [file 10815_2024_3060_MOESM1_ESM.docx]

| Supplemental Table 1 Medicines used for ovulation induction | | | | | |  |
| --- | --- | --- | --- | --- | --- | --- |
| DeDesd Generic name | **Codes** | **Form, strength and pack size** | **Protocol** | **Maximum cycles covered** | **Exposure window around LMP ^a^ (days)** | |
| Clomiphene | 01211R | 50 mg tablet, 10 | 50 mg or 100 mg daily for 5 days | 2 | -81 to 21 | |
| Human chorionic gonadotrophin | 01477R 01579D 01581F 01582G 01583H | 5,000 units/1 mL, 1  500 units/1 mL,3  1,500 units/1 mL, 3  2,000 units/1 mL, 3  500 units/1 mL, 3 | 1 trigger injection of 5,000-10,000 units | 1 | -52 to 21 | |
| Letrozole ^b^ | 08245Y | 2.5 mg tablet, 30 | 1 daily for 5 days | 6 | -197 to 21 | |
| Tamoxifen | 02109B  02110C  01880Y | 10 mg tablet, 60  20 mg tablet, 60  20 mg tablet 30 | 40 or 80 mg daily for 5 days | 3  6  3 | -110 to 21  -197 to 21  -110 to 21 | |

a: Last menstrual period

b: Women with a prior diagnosis of breast cancer were excluded.

As shown above, clomiphene is available on the PBS as a 50 mg tablet, in a pack of 10. To induce ovulation, clomiphene is administered in either a 50 or 100 mg daily dose for five consecutive days. Consequently, each dispensing of clomiphene could last one or two cycles. We considered a clomiphene dispensing to be associated with conception if it was dispensed as early as 81 days before the LMP and as late as 21 days after. We selected 81 days to allow for a woman with oligomenorrhea or amenorrhea a maximum of 31 days to induce a contraceptive pill withdrawal bleed; a 15 day follicular phase; 14 day luteal phase; and added 21 days to account for potential error in the LMP calculation (31 + 15 + 14 + 21 = 81 days). The 21 day period after LMP was also selected due to potential LMP calculation error. The shortest exposure window was calculated for human chorionic gonadotropin (-52 to 21 days) and the longest for letrozole and the 20 mg 60 pack of tamoxifen (-197 to 21 days).

| Supplemental Table 2 International Classiﬁcation of Diseases version 9 with Clinical Modifications (ICD-9-CM) and version 10 with Australian Modiﬁcations (ICD-10-AM) codes used to identify subfertility, complications of pregnancy, maternal pre-existing conditions, and adverse obstetric history | |
| --- | --- |
| DeDesd Description | **Codes** |
|  |  |
| *Previous subfertility* ^a^ | ICD-9-CM: 628, 998.89, V26, V23.0;  ICD-10-AM: N97, N98.1, Z31, Z35.0 |
|  |  |
| *Complications of pregnancy* ^b^ |  |
| Anaemia | ICD-10-AM: O99.00-O99.02 |
| Cervical incompetence | ICD-10-AM: O34.3 |
| Eclampsia | ICD-10-AM: O15 |
| Essential hypertension | ICD-10-AM: O10, O11 |
| Excessive fetal growth | ICD-10-AM: O36.6 |
| Gestational diabetes | ICD-10-AM: O24.4 |
| Labour and delivery complicated by vasa praevia | ICD-10-AM: O69.4 |
| Morbidly adherent placenta | ICD-10-AM: O43.2 |
| Placenta praevia | ICD-10-AM: O44 |
| Placental abruption with haemorrhage | ICD-10-AM: O45.0, O46 |
| Poor fetal growth | ICD-10-AM: O36.5 |
| Postpartum haemorrhage | ICD-10-AM: O72 |
| Preeclampsia | ICD-10-AM: O13, O14 |
| Pre-existing diabetes | ICD-10-AM: O24 (excluding 24.4) |
| Premature labour and delivery | ICD-10-AM: O60 |
| Premature rupture of membranes | ICD-10-AM: O42 |
| Premature separation of placenta | ICD-10-AM: O45.1-O45.9 |
| Shoulder dystocia | ICD-10-AM: O66.0 |
| Threatened abortion | ICD-10-AM: O20 |
| Threatened premature labour | ICD-10-AM: O47.0 |
| Vanishing twin | ICD-10-AM: O31.0-O31.2 |
|  |  |
| *Maternal pre-existing conditions* ^c^ |  |
| Anxiety and depression | ICD-9-CM: 296.2, 296.3, 296.5-296.7, 296.82, 296.89, 296.99, 300, 301.13, 308.3, 311;  ICD-10-AM: F31-F34, F40, F41, F43.8 |
| Asthma | ICD-9-CM: 493; ICD-10-AM: J45-J46 |
| Crohn’s disease | ICD-9-CM: 555; ICD-10-AM: K50 |
| Diabetes | ICD-9-CM: 250;  ICD-10-AM: E09-E11, E13, E14 |
| Epilepsy | ICD-9-CM: 345; ICD-10-AM: G40, G41 |
| Hypertension | ICD-9-CM: 401-405;  ICD-10-AM: I10-I13, I15 |
| Thyroid disorder | ICD-9-CM: 240-246;  ICD-10-AM: E00-E07 |
| Ulcerative colitis | ICD-9-CM: 556; ICD-10-AM: K51 |
|  |  |
| *Adverse obstetric history* ^c^ |  |
| Abortion (spontaneous or medical) | ICD-9-CM: 630-632, 634-639;  ICD-10-AM: O01-O06 |
| Uterine Curettage ^d^ | ICD-9-CM: 69.01-69.09 |
| Ectopic pregnancy | ICD-9-CM: 633; ICD-10-AM: O00 |
| Preterm labour and delivery | ICD-9-CM: 644.21; ICD-10-AM: O60 |
| Stillbirth | ICD-9-CM: V27.1, V27.3, V27.4, V27.6, V27.7; ICD-10-AM: Z37.1, Z37.3, Z37.4, Z37.6, Z37.7 |

a: A 5-year lookback period was used for each conception. All hospital records for this period were covered by ICD-10-AM.

b: The duration of all pregnancies were covered by ICD-10-AM.

c: A 15-year lookback period was used for each conception. Records for this period were covered by ICD-9-CM and ICD-10-AM.

d: Procedure codes in the ICD-10-AM era are expressed as Medicare Benefit Schedule codes, see Appendix 3.

| Supplemental Table 3 Medicare Benefit Schedule codes[19] used to identify subfertility, maternal pre-existing conditions, and adverse obstetric history | |
| --- | --- |
| DeDesd Description | **Codes** |
|  |  |
| *Previous subfertility* |  |
| Assisted Reproductive Technology | 13200-13202 |
| Cycle tracking | 13206 |
| Falloposcopy | 35710-00 |
| Hysterosalpingography | 59712-00 |
| Intracytoplasmic Sperm Injection | 13251 |
| Oocyte retrieval | 13212-00, 13212-01, 13212-02, 13212-03 |
| Ovulation monitoring | 13203 |
| Preparation of eggs | 13218-00, 13218-01, 13218-02 |
| Transfer of ova and sperm | 13215-00, 13215-01 |
| Tubal patency and inflation | 35703-00, 35703-01 |
|  |  |
| *Maternal pre-existing conditions* |  |
| Obesity | 14215-00, 30511, 30512, 30514, 90950-00, 90953-00 |
|  |  |
| *Adverse obstetric history* |  |
| Uterine Curettage | 35640-00, 35640-01, 35640-03, 35643-03 |

| Supplemental Table 4 Anatomic Therapeutic Chemical classification codes used in the Rx-Risk comorbidity index[20] to identify pre-existing maternal chronic conditions in pharmacy data | |
| --- | --- |
| DeDesd Description | **Codes** |
| Anxiety and depression | N05BA01–N05BA12, N05BE01, N06AA01–N06AG02, N06AX03–N06AX11, N06AX13–N06AX18, N06AX21–N06AX26 |
| Epilepsy | N03AA01–N03AX99 |
| Thyroid disorders | H03AA01–H03AA02, H03BA02, H03BB01 |

| Supplemental Table 5 Complications of pregnancy, labour, and delivery for each twin or higher order multiple baby across conception groups ^a^ | | | | | |
| --- | --- | --- | --- | --- | --- |
|  | **Fertile** | **Subfertile untreated** | **Ovulation induction** | **ART** ^b^ | **P-value** |
| Multiple births N | 7,313 | 326 | 404 | 1,532 |  |
|  |  |  |  |  |  |
| Anaemia ^e^ | 604 (8.3%) | 15 (4.6%) | 45 (11.1%) | 110 (7.2%) | 0.006 |
|  |  |  |  |  |  |
| Cervical incompetence ^e^ | 129 (1.8%) | 8 (2.5%) | 17 (4.2%) | 68 (4.4%) | <0.001 |
|  |  |  |  |  |  |
| Urinary Tract Infection ^e,f^ | 309 (4.2%) | 12 (3.7%) | 25 (6.2%) | 50 (3.3%) | 0.056 |
|  |  |  |  |  |  |
| Infection of amniotic sac and membranes ^e^ | 106 (1.4%) | <5 | 6 (1.5%) | 30 (2.0%) | 0.259 |
|  |  |  |  |  |  |
| Vanishing twin ^e^ | 86 (1.2%) | <5 | <5 | 17 (1.1%) | 0.983 |
|  |  |  |  |  |  |
| Preeclampsia/eclampsia ^e, f^ | 897 (12.3%) | 38 (11.7%) | 84 (20.8%) | 210 (13.7%) | <0.001 |
|  |  |  |  |  |  |
| Gestational hypertension without proteinuria ^e^ | 714 (9.8%) | 32 (9.8%) | 52 (12.9%) | 133 (8.7%) | 0.091 |
|  |  |  |  |  |  |
| Gestational diabetes ^e, f^ | 840 (11.5%) | 30 (9.2%) | 48 (11.9%) | 202 (13.2%) | 0.134 |
|  |  |  |  |  |  |
| Morbidly adherent placenta ^e^ | 61 (0.8%) | <5 | <5 | 20 (1.3%) | 0.324 |
|  |  |  |  |  |  |
| Placenta praevia ^f^ | 22 (0.3%) | <5 | <5 | 23 (1.5%) | <0.001 |
|  |  |  |  |  |  |
| Placental abruption ^e, f^ | 107 (1.5%) | 0 | 6 (1.5%) | 33 (2.2%) | 0.026 |
|  |  |  |  |  |  |
| Threatened abortion <20 weeks ^e, f^ | 328 (4.5%) | 22 (6.7%) | 26 (6.4%) | 134 (8.7%) | <0.001 |
|  |  |  |  |  |  |
| Other antepartum haemorrhage ^e, f^ | 457 (6.2%) | 12 (3.7%) | 26 (6.4%) | 172 (11.2%) | <0.001 |
|  |  |  |  |  |  |
| Poor fetal growth ^e^ | 1,044 (14.3%) | 56 (17.2%) | 53 (13.1%) | 249 (16.3%) | 0.094 |
|  |  |  |  |  |  |
| Excessive fetal growth ^e^ | 16 (0.2%) | <5 | <5 | 10 (0.7%) | 0.028 |
|  |  |  |  |  |  |
| Threatened preterm labour <37 weeks ^e, f^ | 1519 (20.8%) | 54 (16.6%) | 71 (17.6%) | 351 (22.9%) | 0.017 |
|  |  |  |  |  |  |
| Prelabour rupture of membranes ^e, f^ | 1155 (15.8%) | 42 (12.9%) | 74 (18.3%) | 269 (17.6%) | 0.075 |
|  |  |  |  |  |  |
| Complications of labour and delivery | |  |  |  |  |
|  |  |  |  |  |  |
| vasa praevia ^e^ | <5 | <5 | 0 | 6 (0.4%) | <0.001 |
|  |  |  |  |  |  |
| Prolapsed cord ^f^ | 64 (0.9%) | <5 | 0 | 12 (0.8%) | 0.301 |
|  |  |  |  |  |  |
| Emergency caesarean ^f^ | 2,242 (30.7%) | 101 (31.0%) | 113 (28.0%) | 583 (38.1%) | <0.001 |
|  |  |  |  |  |  |
| Postpartum haemorrhage (PPH) e,f  -vaginal delivery  -elective caesarean  -emergency caesarean | 2893 (39.6%)  859 (31.4%)  818 (35.0%)  1216 (54.2%) | 115 (35.3%)  32 (45.1%)  45 (29.2%)  38 (37.6%) | 125 (30.9%)  36 (35.3%)  45 (23.8%)  44 (38.9%) | 557 (36.4%)  101 (33.8%)  182 (28.0%)  274 (47.0%) | <0.001  0.073  <0.001  <0.001 |
|  |  |  |  |  |  |
| Preterm birth - live births only ^f^  <32 weeks  32-36 weeks | 713 (10.0%)  3549 (49.7%) | 29 (9.0%)  176 (54.3%) | 44 (11.1%)  193 (48.6%) | 183 (12.3%)  882 (59.1%) | <0.001 |
|  |  |  |  |  |  |
| Low birthweight – live births only ^f^  <1500g  1500-<2500g | 590 (8.3%)  3138 (43.9%) | 21 (6.5%)  158 (48.8%) | 38 (9.6%)  167 (42.1%) | 161 (10.8%)  740 (49.6%) | <0.001 |
|  |  |  |  |  |  |
| Perinatal death ^a,^ ^f, g, h^  -stillbirth | 234 (3.2%)  165 (2.3%) | <5  <5 | 11 (2.7%)  7 (1.7%) | 54 (3.5%)  40 (2.6%) | 0.097  0.145 |
|  |  |  |  |  |  |

^a^ denominator = babies.

^b^ Assisted Reproductive Technology.

^d^ Determined from pharmacy claims.

^e^ Determined from hospital records.

^f^ Determined from Midwives’ records.

^g^ Determined from death records.

^h^ Perinatal death refers to a death between 20 weeks gestation and 28 days following birth.

Where more than one superscript – determined from combined results of more than one dataset.

**Supplemental Table 6** Effect of missing data on risk ratios for preterm birth, low birthweight and perinatal death for ART, OI and subfertile untreated singleton births compared with fertile natural conceptions. RR estimates are calculated using a basic Poisson model with robust SEs adjusted for key covariates with no missing data on the whole dataset and the reduced dataset (where cases with missing private health insurance status and/or marital status and/or SES are excluded). Differences in RR estimates are solely due to missing data

|  | N as per crude model (no missing data)  (all basic covariates available) | | | N as per fully adjusted model (cases with missing PHI/marital status/SES are excluded) | |
| --- | --- | --- | --- | --- | --- |
|  | N | Crude RR (95% CI) | RR (95% CI) Basic^3^ | N | RR (95% CI) Basic^3^ |
| **Preterm Birth^1^** |  |  |  |  |  |
| *Fertile* | 17809/298426 | 1.00 (reference) | 1.00 (reference) | 17571/292296 | 1.00 (reference) |
| Subfertile untreated | 878/11237 | 1.31 (1.23-1.40) | 1.30 (1.22-1.39) | 872/11104 | 1.30 (1.21-1.39) |
| Ovulation induction | 291/3652 | 1.34 (1.20-1.49) | 1.34 (1.20-1.49) | 288/3612 | 1.33 (1.19-1.48) |
| ART | 915/8626 | 1.78 (1.67-1.89) | 1.68 (1.57-1.79) | 909/8517 | 1.68 (1.57-1.79) |
|  |  |  |  |  |  |
| **Low Birthweight^1^** |  |  |  |  |  |
| *Fertile* | 12961/298426 | 1.00 (reference) | 1.00 (reference) | 12766/292296 | 1.00 (reference) |
| Subfertile untreated | 574/11237 | 1.18 (1.08-1.28) | 1.28 (1.18-1.39) | 570/11104 | 1.28 (1.18-1.39) |
| Ovulation induction | 186/3652 | 1.17 (1.02-1.35) | 1.24 (1.08-1.43) | 183/3612 | 1.23 (1.06-1.41) |
| ART | 576/8626 | 1.54 (1.42-1.67) | 1.52 (1.40-1.65) | 575/8517 | 1.52 (1.40-1.66) |
|  |  |  |  |  |  |
| **Perinatal death^2^** |  |  |  |  |  |
| *Fertile* | 2284/300285 | 1.00 (reference) | 1.00 (reference) | 2215/294100 | 1.00 (reference) |
| Subfertile untreated | 103/11319 | 1.20 (0.98-1.46) | 1.23 (1.01-1.50) | 99/11182 | 1.21 (0.99-1.49) |
| Ovulation induction | 26/3671 | 0.93 (0.63-1.37) | 0.96 (0.65-1.41) | 26/3631 | 0.98 (0.66-1.44) |
| ART | 103/8711 | 1.56 (1.28-1.89) | 1.57 (1.28-1.92) | 103/8602 | 1.61 (1.31-1.97) |

^1^ Number of preterm and low birth weight singletons are shown with a denominator of singleton livebirths.

^2^ Number of perinatal deaths are shown with a denominator of livebirths and stillbirths.

^3^ Basic adjusted Poisson model includes year of birth group, maternal age group, parity group, ethnic origin, smoking, pre-existing diabetes, essential hypertension.
